# Research status and trends of functional magnetic resonance imaging technology in the field of acupuncture: a bibliometric analysis over the past two decades

**DOI:** 10.3389/fnins.2025.1489049

**Published:** 2025-01-30

**Authors:** Zhongke Wang, Lu Chen, Tianyu Jiang, Qi Zhang, Jinying Zhao, Fuchun Wang

**Affiliations:** Department of Acupuncture and Tuina, Changchun University of Traditional Chinese Medicine, Changchun, China

**Keywords:** acupuncture, fMRI, bibliometric analysis, Citespace, VOSviewer

## Abstract

**Objective:**

Although fMRI has been widely used in the field of acupuncture. However, the literature analysis in this field still has significant differences. This study summarizes the current status of functional magnetic resonance imaging (fMRI) in the field of acupuncture and moxibustion and predicts its future trends through Web of Science bibliometric analysis.

**Methods:**

This study uses “fMRI” and “acupuncture” as keywords to search for literature related to functional magnetic resonance imaging (fMRI) in acupuncture research in the Web of Science Core Collection database from January 1, 2004, to April 30, 2024. Visualization analyses were conducted using Citespace (version 6.3 R1) and VOSviewer (version 1.6.20). Citespace was employed to analyze annual publications, countries, institutions, keywords, and co-cited references. VOSviewer was used to analyze authors and co-cited authors, as well as journals and co-cited journals.

**Results:**

From 2004 to 2024, a total of 967 publications were retrieved, of which 557 were included after screening. Despite annual fluctuations, the overall trend shows an increase. China and the Chinese Academy of Sciences are the countries and institutions with the highest number of publications, with Tian, J being the author with the most publications, and Kong, J having the highest Co-citation frequency. The article by Dhond, RP, published in 2008, has the highest Co-citation frequency among the co-cited literature. Evidence-based Complementary and Alternative Medicine is the journal with the most publications, while Neuroimage is the co-cited journal with the highest citation frequency. Keyword co-occurrence and burst reveal the main research hotspots, including the diversity of intervention methods, cortical activation, mechanisms related to pain-associated diseases, and brain-related diseases. Keyword burst detection reflects emerging trends, including meta-analysis and systematic reviews, the relationship between ischemic stroke and women, and the connection between mild cognitive impairment and prevention.

**Conclusion:**

This study employs bibliometric methods to explore the current status, research hotspots, and frontier issues regarding the application of functional magnetic resonance imaging (fMRI) technology in the field of acupuncture, providing new perspectives and directions for acupuncture fMRI research.

## 1 Introduction

As a green and effective therapeutic method, acupuncture has a history of over 3,000 years in China and is widely applied in the treatment of neurological, endocrine, and respiratory systems (Wen et al., 2021). Despite its study since the 18th century and significant progress in clinical practice as well as in the understanding of acupoints and meridians, the clinical efficacy and physiological and biological mechanisms of acupuncture remain incompletely elucidated (Zhuang et al., 2013). Functional magnetic resonance imaging (fMRI), a leading non-invasive neuroimaging technique, offers excellent spatial and temporal resolution. Since the 1990s, fMRI has been applied in acupuncture research, including studies on the characterization of needling stimulation and brain responses before and after acupuncture (Napadow et al., 2008). Currently, fMRI is frequently used to evaluate the clinical effects of acupuncture interventions or to investigate its mechanisms of influence on the central nervous system (Yan et al., 2022).

This study employs Citespace and VOSviewer for visualization analysis. Citespace is used to analyze annual publications, countries, institutions, keywords, and co-cited references, while VOSviewer is applied to examine authors and co-cited authors, journals, and co-cited journals. The aim is to explore the application of fMRI in the field of acupuncture and conduct an in-depth analysis of its current research status and trends, thereby providing guidance and a foundation for further studies on the application of fMRI in acupuncture.

## 2 Materials and methods

### 2.1 Data source and retrieval strategy

The literature utilized in this study was sourced from the SCI-EXPANDED database within the Web of Science Core Collection, which is accessible at Changchun University of Traditional Chinese Medicine Library. The retrieval period spanned from January 1, 2004, to April 30, 2024. The retrieval process was concluded on May 26, 2024. The database search strategy encompassed the utilization of keywords “acupuncture” and “fMRI.”

### 2.2 Criteria for inclusion and exclusion of literature

Inclusion criteria: article or review article. The language is English, without any restrictions on the country of publication.

Exclusion criteria: editorial material, meeting abstract, proceeding paper, letter, correction, retracted publication, early access, book chapters, and literature unrelated to the topic.

### 2.3 Data collection and analysis

The data should be converted to “txt” format, named “download_*.txt,” and subsequently imported into Citespace (version 6.3 R1) and VOSviewer (version 1.6.20) for conducting data analysis on selected literature. The analysis includes information such as annual publications, journals, countries, institutions, authors, keywords, authors and their Co-citations, journal and their Co-citations, and references Co-citations. In CiteSpace, the parameter settings are as follows: (1) The time range is set from 2004 to 2024 with a yearly interval; (2) Only one node type is selected at a time; (3) The g-index selection *k*-value is set to 25; (4) The Top-N option is set to 50. In the Citespace network knowledge map, nodes represent the informational units of the analyzed items, with node size indicating the frequency of occurrence. The color and width of the “annual rings” within nodes reflect the timing and quantity of occurrences for the analyzed items, while a purple outer ring signifies high betweenness centrality within the overall network. The thickness of the connecting lines illustrates the strength of the relationships between different analyzed items.

## 3 Results

### 3.1 Document screening situation

**Figure 1:**
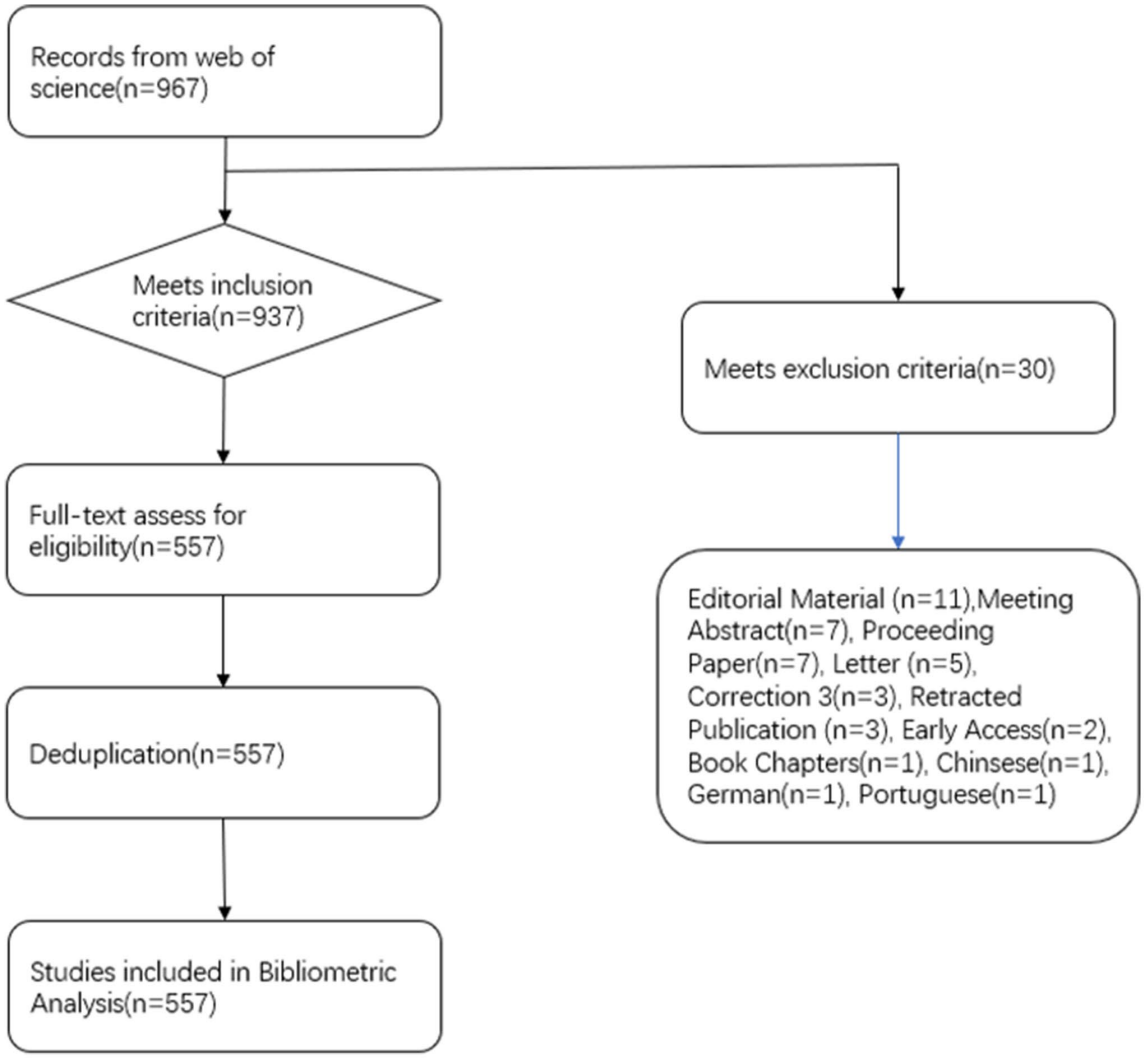
Flow chart of literature screening.

We acquired a total of 967 sources, consisting of 838 articles, 103 review articles, 11 editorial materials, seven meeting abstracts, seven proceeding papers, five letters, and three corrections. Additionally, there were also three retracted publications and two early-access manuscripts. Furthermore, we identified one book chapter in our search. In terms of language, there are 964 articles written in English, one in Chinese, one in German, and one in Portuguese. After applying inclusion criteria, we selected a total of 937 articles and excluded an additional 380 articles that were deemed irrelevant to the topic. Therefore, a total of 557 records were included for further visualization and analysis in this study. Quantitative methods were employed to analyze the current status and trends of functional magnetic resonance imaging technology in acupuncture, following the process depicted in Figure 1. The task of conducting database searches and screening literature was assigned to two researchers, Zhongke Wang, and Lu Chen, while the responsibility of handling potential disputes or disagreements were undertaken by senior researcher Fuchun Wang.

### 3.2 Analysis of annual publications

**Figure 2:**
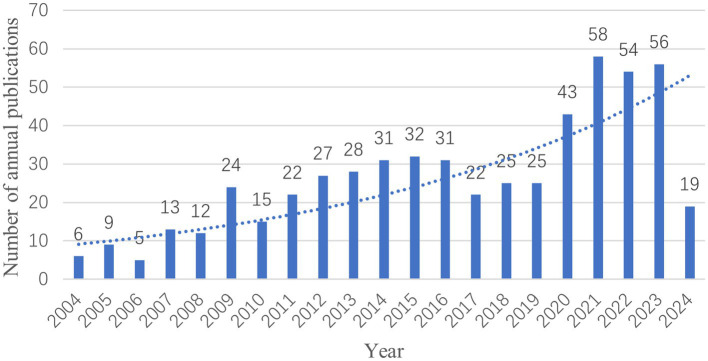
The number of fMRI in the field of acupuncture for annual publications.

The number of publications per year is shown in Figure 2, which can be divided into three stages. The first stage, from 2004 to 2010, saw slight fluctuations in the number of publications but exhibited a continuous growth trend, with a peak of 24 articles in 2009. However, the number of publications decreased to 15 articles in 2010. The second stage, from 2010 to 2017, was characterized by a steady increase in publication volume, although the rate of increase was relatively slow, with a decline to 22 articles in 2017. The third stage, from 2017 to 2024, witnessed a gradual increase in publications, particularly between 2019 and 2021, where the growth rate was notably higher, peaking in 2021. Based on data available up to April 2024, it is expected that the number of publications will continue to remain relatively high in the future.

### 3.3 Analysis of national cooperative networks

**Figure 3:**
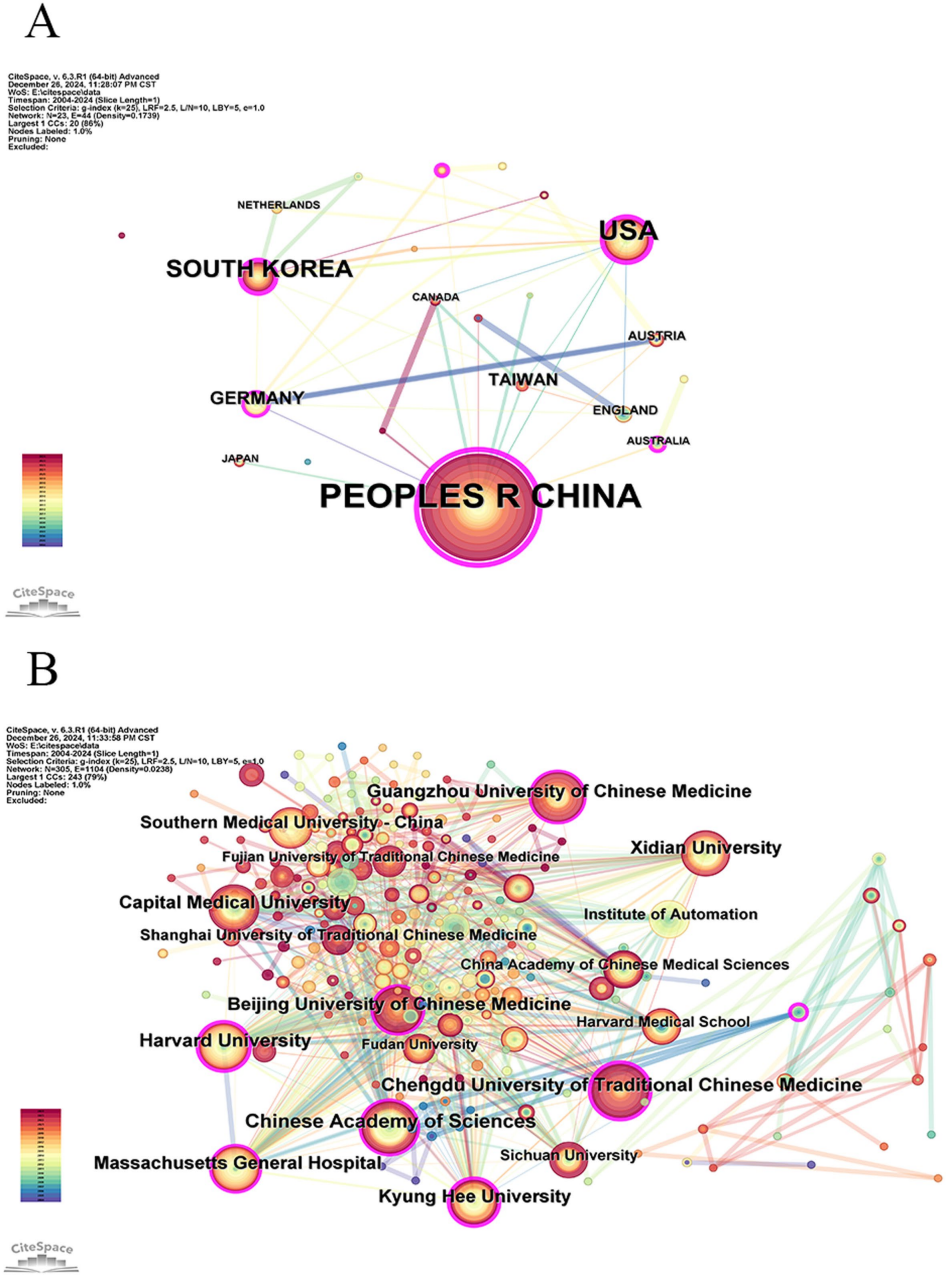
National cooperative networks **(A)** and institutional cooperative networks **(B)**.

The “Country” node should be selected for conducting a visual analysis of the countries mentioned in the literature using a knowledge graph. The study yielded a total of 23 nodes and 44 links, spanning across 23 countries or regions. The top five countries in terms of national publications are Peoples R China (422 articles), USA (116 articles), South Korea (62 articles), Germany (22 articles), and Taiwan (China) (16 articles). According to the centrality ranking, the top five countries are Peoples R China (0.79), USA (0.31), the Republic of Korea (0.2), Australia (0.16), and Switzerland (0.16, Table 1). The rankings of Peoples R China, USA, and South Korea consistently occupy the top three positions based on considerations of publication frequency and centrality. As shown in Figure 3A, the formation of China, USA, and South Korea as three cores signifies the presence of strong internal connections among these countries. The density value of 0.1739 suggests a relatively moderate level of inter-country cooperation.

**Table 1:** Top 10 authors related to fMRI in the field of acupuncture.

Rank	Publications	Country/region	Rank	Centrality	Country/region
1	422	Peoples R China	1	0.79	Peoples R China
2	116	USA	2	0.31	USA
3	62	South Korea	3	0.20	South Korea
4	22	Germany	4	0.16	Australia
5	16	Taiwan (China)	5	0.16	Switzerland

### 3.4 Network analysis of institutional collaboration

The “Institution” node should be selected to visualize and analyze the knowledge graph of institutions mentioned in the literature. The network consists of 305 nodes and 1,104 links, encompassing a total of 305 distinct institutions. Table 2 lists the top five institutions in terms of publication and centrality. Chinese Academy of Sciences takes the lead with the highest research output (74 articles), followed by Harvard University (65 articles) and Chengdu University of Traditional Chinese Medicine (65 articles), which are tied for second place. Massachusetts General Hospital ranks fourth (60 articles), while Beijing University of Chinese Medicine and Xidian University share fifth place with 58 articles each. In terms of centrality, the institutions are ranked as follows: Kyung Hee University (0.35), Harvard University (0.23), Chengdu University of Traditional Chinese Medicine (0.21), University of California System (0.2), and Massachusetts General Hospital (0.19, Table 3). The nodes of the Beijing University of Chinese Medicine, Chinese Academy of Sciences, Chengdu University of Traditional Chinese Medicine, and other institutions are prominently large in Figure 3B, indicating their central position and strong connections within their respective organizations. However, the density value is 0.0238, indicating a lack of cohesive collaboration among various institutions. Moreover, China boasts a substantial number of institutions engaged in fMRI research applied to acupuncture.

**Table 2:** Top five institution by publications.

Rank	Publications	Institution
1	74	Chinese Academy of Sciences
2	65	Harvard University
3	65	Chengdu University of Traditional Chinese Medicine
4	60	Massachusetts General Hospital
5	58	Beijing University of Chinese Medicine
6	58	Xidian University

**Table 3:** Top five institution by centrality.

Rank	Centrality	Institution
1	0.35	Kyung Hee University
2	0.23	Harvard University
3	0.21	Chengdu University of Traditional Chinese Medicine
4	0.20	University of California System
5	0.19	Massachusetts General Hospita

### 3.5 Analysis of the authors and co-cited authors

**Figure 4:**
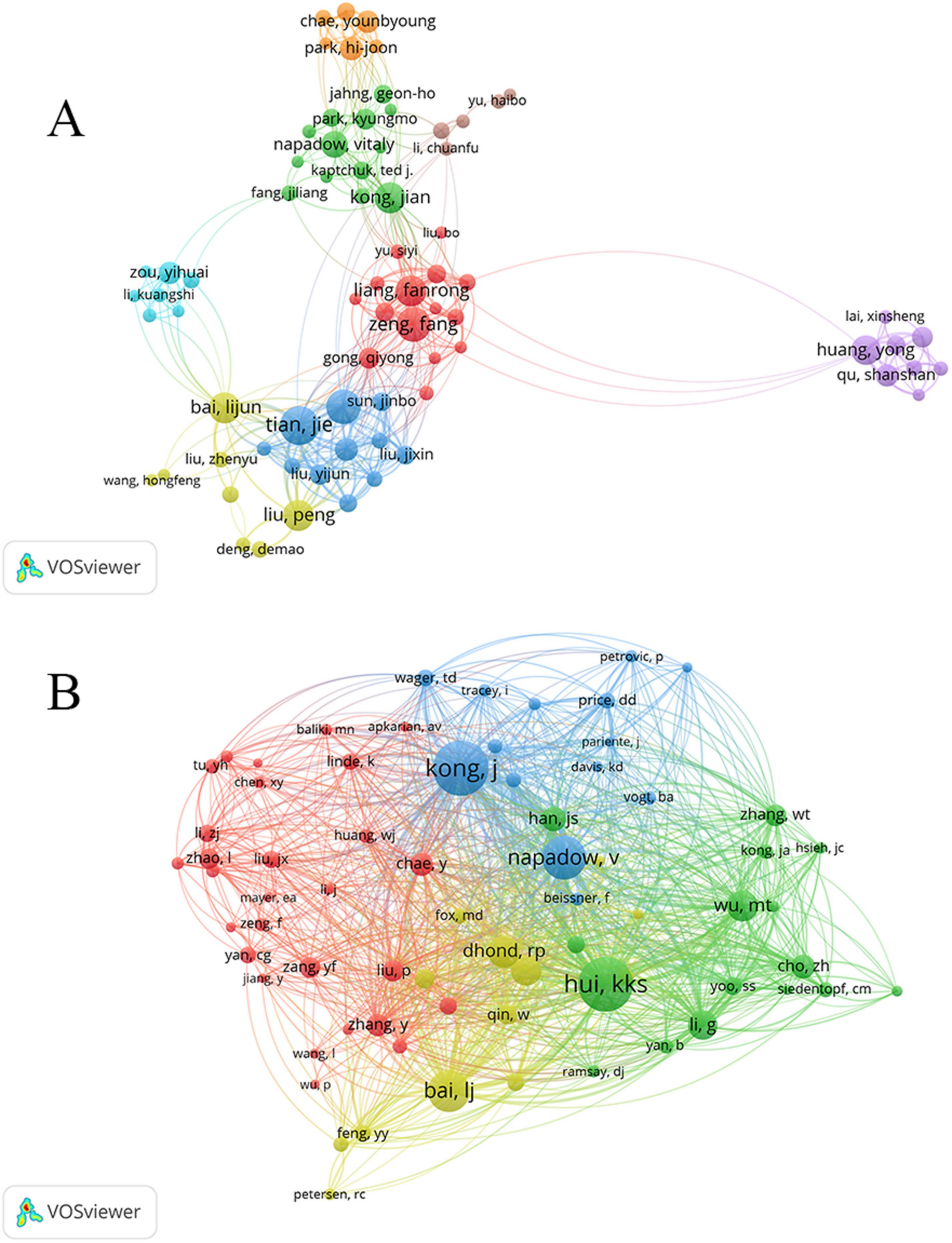
Map of the authors **(A)** and co-cited authors **(B)**.

The author collaboration network was generated using VOSviewer, which includes 2,706 authors who published 557 articles. In Figure 4A, authors with more than 7 publications are displayed. Each node represents an author, with the size of the node positively correlated with the number of publications. The connections between nodes represent collaborative relationships, with thicker lines indicating stronger cooperation. In the context of fMRI research related to acupuncture, the top 10 authors are shown in Table 4. Tian, J ranks first (*n* = 36), followed by Qin, W; Zeng, F; Bai, LJ; Kong, J; and Liang, FR. Among them, Kong, J and Liang, FR are tied for fifth place (*n* = 26). Analysis of the author collaboration network using VOSviewer reveals the formation of 8 clusters, with each cluster consisting of multiple authors engaged in long-term collaboration.

**Table 4:** Top five authors and co-cited authors.

Rank	Publications	Author	Rank	Citations	Cited authors
1	36	Tian, J	1	360	Kong, J
2	30	Qin, W	2	350	Hui, KKS
3	30	Zeng, F	3	259	Napadow, V
4	26	Bai, LJ	4	241	Bai, LJ
5	26	Kong, J	5	162	Dhond, RP
6	26	Liang, FR			

The total number of co-cited authors is 10,734, among whom 12 authors have received more than 100 citations. Figure 4B illustrates the authors with citation frequencies exceeding 30, while Table 4 showcases the top five most frequently cited authors. Among them, Kong, J has received the highest number of citations with a count of 360. Following closely are Hui, KKS with 350 citations, Napadow, V with 259 citations, Bai, LJ with 241 citations, and Dhond, RP with 162 citations.

### 3.6 Analysis of journals and co-cited journals

**Figure 5:**
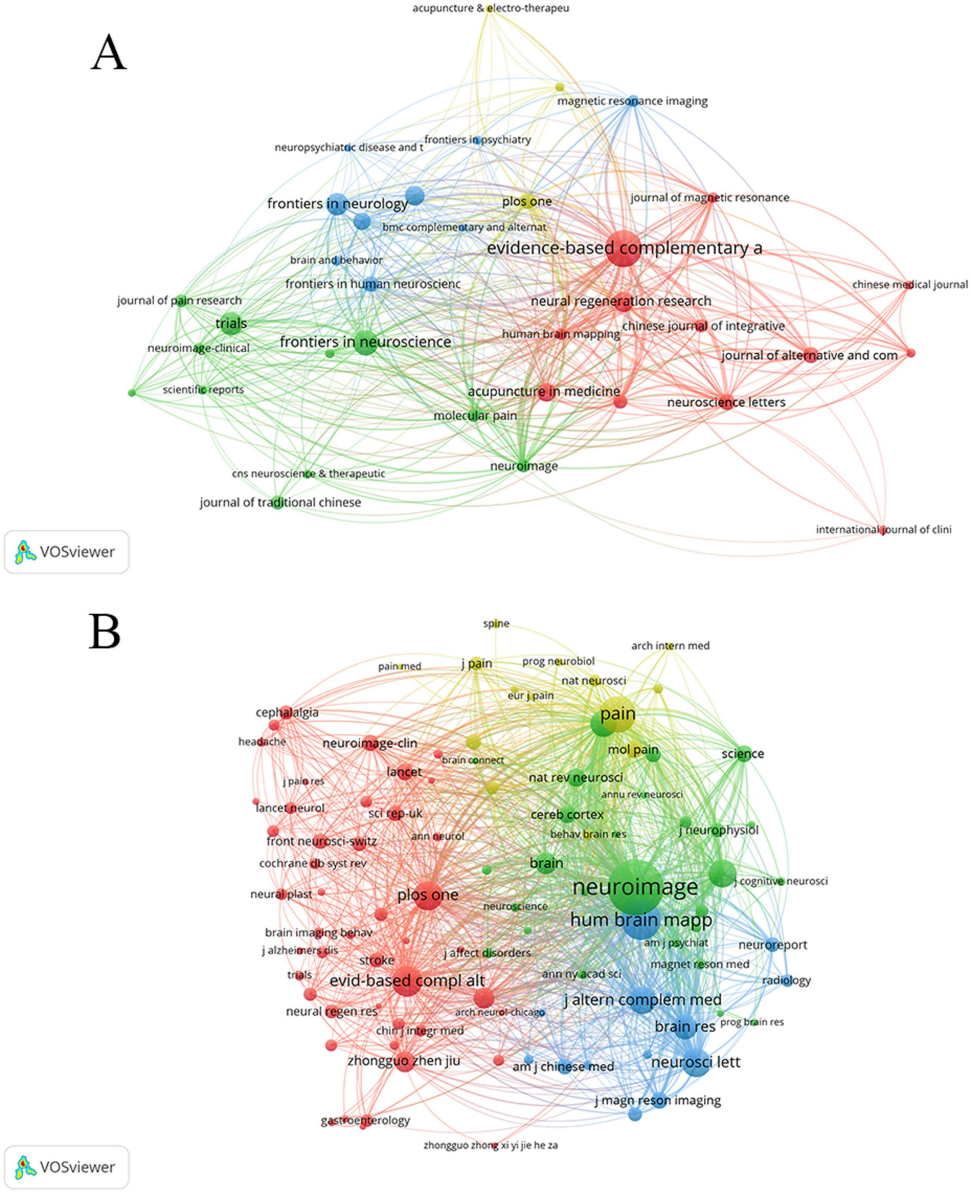
Map of journals **(A)** and co-cited journals **(B)**.

Journal and journal Co-citation analysis was conducted using VOSviewer. Publications on the application of fMRI in the field of acupuncture were published in over 138 journals, with Figure 5A displaying the journals that appeared more than four times. Table 5 presents the top 10 most popular academic journals in the field, with Evidence-based Complementary and Alternative Medicine having the highest number of publications (*n* = 58), followed by Frontiers in Neuroscience (*n* = 30), Trials (*n* = 25), Frontiers in Neurology (*n* = 24), and Neural Regeneration Research (*n* = 20). Furthermore, Neuroscience Letters and Plos One are tied for tenth place. According to Journal Citation Reports 2023, Neural Regeneration Research has achieved a high impact factor (IF =5.9). Unfortunately, the majority of journals have an impact factor below five, and JCR categories are primarily concentrated within Q3.

**Table 5:** Top 10 journal and co-cited journal.

Rank	Count	Journal	IF/JCR (2023)	Rank	Co-citation	Co-cited journal	IF/JCR (2023)
1	58	Evidence-based Complementary and Alternative Medicine	2.65/Q3	1	1,618	Neuroimage	4.7/Q1
2	30	Frontiers in Neuroscience	3.2/Q2	2	885	hum brain mapp	3.5/Q2
3	25	Trials	2.0/Q3	3	766	pain	5.9/Q1
4	24	Frontiers in Neurology	2.7/Q2	4	580	Evidence-based Complementary and Alternative Medicine	2.65/Q3
5	20	Neural Regeneration Research	5.9/Q2	5	519	Plos One	2.9/Q2
6	19	Medicine	1.3/Q3	6	502	Neuroscience letters	2.5/Q3
7	18	Acupuncture in Medicine	2.4/Q3	7	479	Proceedings of The National Academy of Sciences of The United States of America	9.4/Q1
8	16	Neural Plasticity	3.0/Q3	8	469	Journal of Alternative and Complementary Medicine	2.3/Q3
9	14	Journal of Alternative and Complementary Medicine	2.3/Q3	9	448	J Neurosci	4.4/Q1
10	14	Neuroscience letters	2.5/Q3	10	381	Brain Research	2.7/Q3
11	14	Plos One	2.9/Q2				

The total number of cited journals is 3,278, out of which 6 journals have citation counts exceeding 500. Figure 5B shows that journals are cited more than 40 times. As shown in Table 5, the top 10 academic journals are Neuroimage (Co-citation = 1,618), followed by Hum Brain Mapp (Co-citation = 885), Pain (Co-citation = 766), Evidence-based Complementary and Alternative Medicine (Co-citation = 580), Plos One (Co-citation = 519) and Neuroscience letters (Co-citation = 502). Additionally, Proceedings of The National Academy of Sciences of The United States of America has the highest impact factor (IF = 9.4), followed by pain (IF = 5.9) and Neuroimage (IF = 4.7), while the remaining journals have impact factors below 5.

### 3.7 Analysis of co-cited references

**Figure 6:**
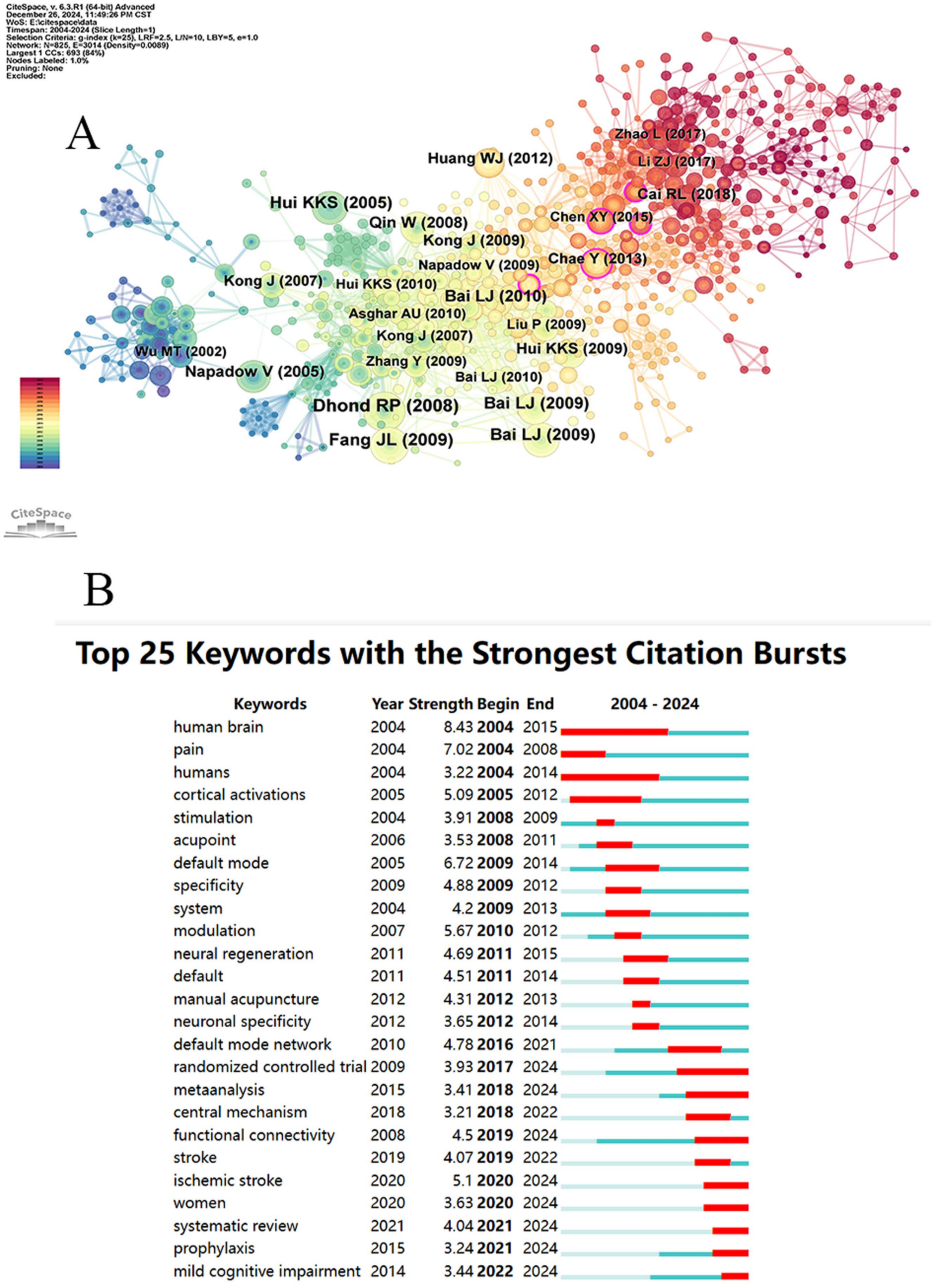
Map of co-cited references **(A)** and Top 25 keywords with the strongest citation bursts **(B)**.

A Co-citation analysis of references was conducted using the “Reference” feature, as shown in Figure 6A. The analysis revealed a network consisting of 825 nodes and 3,014 links, with a network density of 0.0089, indicating relatively weak connections between these 825 cited references. According to Table 6, the top five most frequently cited references include Dhond RP, 2008; Fang JL, 2009; Bai LJ, 2009; Hui KKS, 2005; and Bai LJ, 2009, which are considered representative. In terms of the centrality ranking of cited references, the top five are You YB, 2013 (0.21); Wang XY, 2016 (0.13); Chae Y, 2013 (0.11); Chen XY, 2015 (0.11); Kong J, 2009 (0.1); and Li ZJ, 2016 (0.1), as shown in Table 7. Notably, Kong J, 2009, and Li ZJ, 2016 (0.1) are tied for fifth place. It is worth mentioning that despite these two references being published later, Kong J and Li ZJ are highly representative of the application of fMRI in the field of acupuncture.

**Table 6:** Top five cited reference by frequency.

Rank	Freq	Cited reference
1	49	Acupuncture modulates resting state connectivity in default and sensorimotor brain networks
2	46	The salient characteristics of the central effects of acupuncture needling: limbic-paralimbic-neocortical network modulation
3	40	Acupuncture modulates spontaneous activities in the anticorrelated resting brain networks
4	38	The integrated response of the human cerebro-cerebellar and limbic systems to acupuncture stimulation at ST 36 as evidenced by fMRI
5	38	Time-varied characteristics of acupuncture effects in fMRI studies

**Table 7:** Top five cited reference by centrality.

Rank	Centrality	Cited reference
1	0.21	Altered hub configurations within default mode network following acupuncture at ST36: a multimodal investigation combining fMRI and MEG
2	0.13	Repeated acupuncture treatments modulate amygdala resting state functional connectivity of depressive patients
3	0.11	Inserting needles into the body: a meta-analysis of brain activity associated with acupuncture needle stimulation
4	0.11	The modulation effect of longitudinal acupuncture on resting state functional connectivity in knee osteoarthritis patients
5	0.10	Functional neuroanatomical investigation of vision-related acupuncture point specificity--a multisession fMRI study
6	0.10	Altered periaqueductal gray resting state functional connectivity in migraine and the modulation effect of treatment

### 3.8 Co-occurrence analysis of keywords

**Figure 7:**
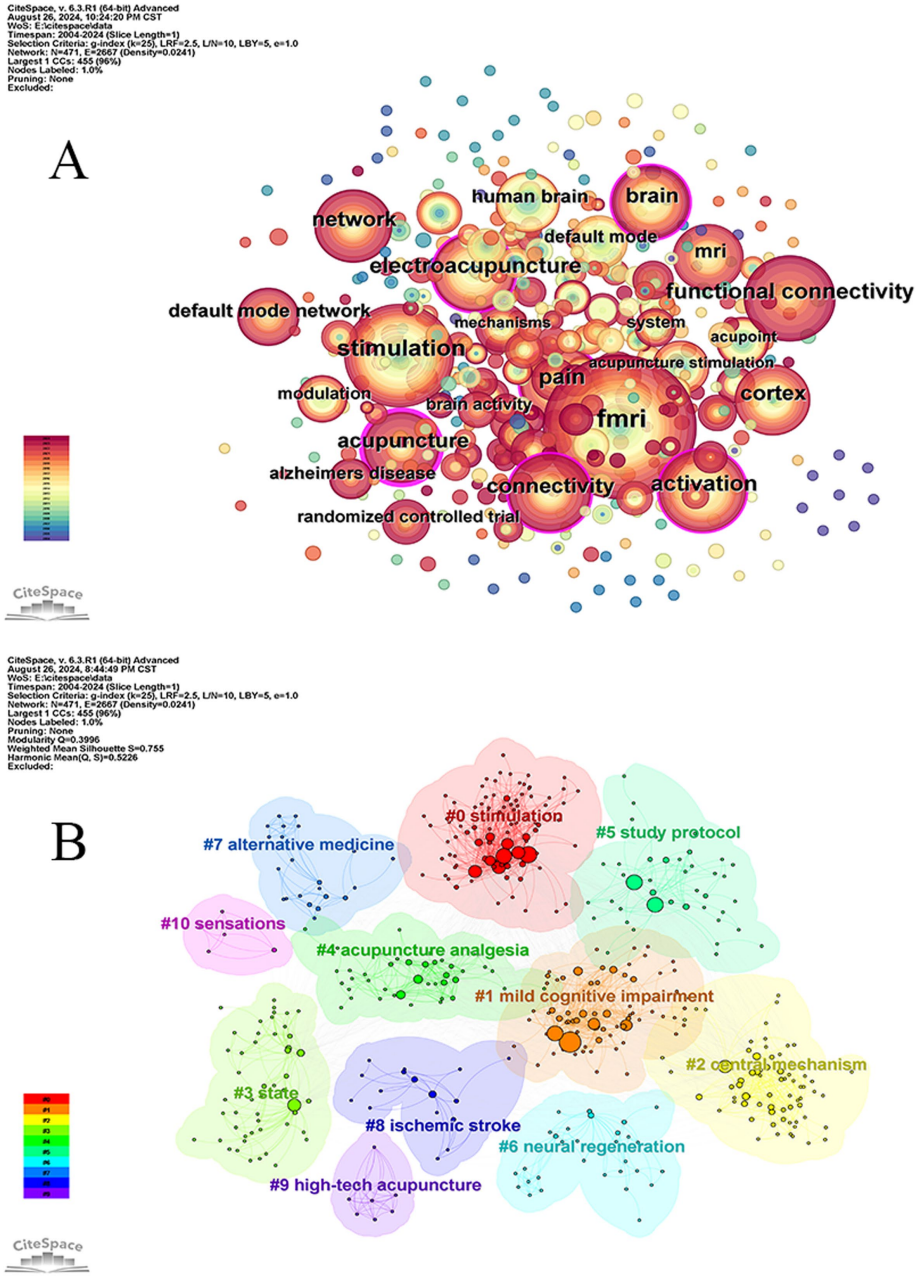
Map of co-occurring keywords **(A)** and keyword clustering **(B)**.

The visualization analysis graph of the keyword co-occurrence network in this study consisted of 471 nodes and 2,667 links, resulting in a network density of 0.0241 (Figure 7A). The top 10 keywords, listed in descending order, include fMRI (223), stimulation (126), electroacupuncture (104), activation (104), pain (102), functional connectivity (101), connectivity (78), acupuncture (77), brain (76) and cortex (Zhang et al., 2021) as shown in Table 8. The frequency analysis indicates that fMRI is currently a research hotspot, with acupuncture having the highest centrality (0.24). A centrality value >0.1 suggests that this node has the highest attention and influence within the research field, representing a key area of focus in the domain.

**Table 8:** Top 10 keywords.

Rank	Freq	Keywords	Rank	Centrality	Keywords
1	223	fMRI	1	0.24	acupuncture
2	126	stimulation	2	0.14	electroacupuncture
3	104	electroacupuncture	3	0.13	connectivity
4	104	activation	4	0.11	pain
5	102	pain	5	0.11	brain
6	101	functional connectivity	6	0.10	activation
7	78	connectivity	7	0.10	MRI
8	77	acupuncture	8	0.09	fMRI
9	76	brain	9	0.08	cortex
10	73	cortex	10	0.08	default mode network
				0.08	human brain

### 3.9 Analysis of keyword clustering

The keywords about acupuncture research utilizing fMRI were examined through the implementation of the “K” clustering-LLR algorithm. Figure 7B results demonstrated that the modularity Q value surpassed the threshold of 0.3, reaching 0.3996, thereby indicating a distinct and substantial clustering structure. Additionally, the mean silhouette value for clustering was determined to be 0.755, implying that this particular clustering method is both rational and dependable. Based on different cluster labels, a total of 11 clusters were obtained: #0 stimulation, #1 mild cognitive impairment, #2 central mechanism, #3 state, #4 acupuncture analgesia, #5 research design, #6 neural regeneration, #7 alternative medicine, #8 ischemic stroke and #9 high-tech acupuncture as well as #10 sensation. The labels #0, #7, and #9 primarily pertain to intervention methods, indicating a diverse range of approaches for interventions and highlighting the need for further research in the field of alternative medicine on this specific topic. The primary focus of Labels #1 and #8 lies in brain-related diseases, while Labels #2, #3, #4, #6, and #10 are associated with research mechanisms. This suggests that scholars have begun to direct their attention toward the action mechanisms of acupuncture.

### 3.10 Analysis of keywords with the strongest citation bursts

Through burst analysis of the selected literature keywords, 25 burst terms were identified (Figure 6B). Early research primarily focused on foundational concepts such as “human brain,” “pain,” “humans,” “stimulation,” and “cortical activation,” with studies on the human brain being the earliest and most sustained. Mid-term research emphasized mechanism exploration, with keywords like “default mode,” “specificity,” “modulation,” and “neural regeneration” emerging. Later, the focus shifted to randomized controlled trials (RCT) to explore disease mechanisms and summarize the applications of fMRI in the field of acupuncture. During this phase, keywords such as “randomized controlled trial,” “meta-analysis,” “functional connectivity,” “ischemic stroke,” “women,” “systematic review,” “prophylaxis,” and “mild cognitive impairment” emerged, which remain significant today. These keywords are expected to become key areas of research focus and trends in the future.

## 4 Discussion

### 4.1 General information

This study employed Citespace and VOSviewer software to conduct a visual analysis of fMRI research in the field of acupuncture, encompassing a total of 557 articles. Through analyses of annual publications, collaboration networks, and Co-citation patterns, the latest advancements in fMRI research within the field of acupuncture were unveiled.

The distribution of annual publications shows that research on fMRI in acupuncture can be divided into three phases. The period from 2004 to 2010 is considered the initiation phase, with a relatively low number of publications and limited research in the field. During this phase, studies mainly focused on the mechanisms of acupuncture stimulation and pain. From 2010 to 2017, there was a steady upward trend in publications. Research during this phase mainly concentrated on neuroregeneration, neural specificity, and the default network. Since 2017, there has been a notable increase in the number of publications, with broader attention given to this area of research. Studies in this later phase have focused on randomized controlled trials, meta-analysis, systematic reviews, ischemic stroke in women, and the prevention of mild cognitive impairment. As the search strategy was conducted with a cutoff date of April 30, 2024, the publication volume for 2024 is lower. However, with 19 publications already published, it is expected that the publication volume for 2024 will at least match or even exceed that of 2023.

The Peoples R China has the highest number of publications, significantly outpacing the USA and other countries and regions, and holds the highest centrality, indicating a deep engagement with research in this field. This is likely related to China’s role as the birthplace of acupuncture. The USA and South Korea rank second and third in terms of publication volume, and notably, they also rank second and third in centrality, reflecting their broader research activities and the publication of influential articles. Germany and Taiwan (China) are ranked fourth and fifth in terms of publication volume. Compared to these four countries, Australia and Switzerland have published fewer studies on fMRI in acupuncture, but both countries show similar performance in centrality. Furthermore, there is limited exchange between countries, highlighting the need to strengthen international collaboration.

According to the collaborative network of institutions, it is evident that China holds four out of the top five positions in terms of publication volume. Similarly, when considering centrality, four out of the top five institutions are from China. These institutions primarily comprise research institutes or universities as their core teams. Kyung Hee University ranks fifth in terms of publication volume, yet it possesses a high centrality score of 0.18 > 0.1, indicating its significant research output and considerable influence in this field. Although Harvard University’s publication volume did not rank among the top five, its centrality score of 0.16 > 0.1 suggests that despite a relatively lower number of publications, their exceptional quality remains evident.

The collaborative network analysis results conducted by the author, Tian, J has emerged as the most prolific researcher, demonstrating an impressive publication count of 36. Closely following are Qin, W, Zeng, F, Bai, LJ, Kong, J and Liang FR. After analyzing the results of co-cited authors, it is found that Kong, J has the highest number of citations, followed by Hui, KKS, Napadow, V, Bai, LJ, and Dhond, RP.

Tian, J and Qin, W have mostly focused on acupoint specificity. They focused on changes in the brain area after needling acupuncture points. Zeng, F and Liang, FR have mostly focused on the central mechanisms of acupuncture for the treatment of diseases. Bai, LJ’s study focused on acupoint specificity, followed by more research on brain area changes after acupuncture treatment of disease. Much of Kong, J’s research has focused on the central mechanisms and brain region changes in the acupuncture treatment of disease. Hui, KKS research focused on brain networks and cortical plasticity. Napadow, V. and Dhond, RP’s research focused on brain networks and neuroplasticity.

The analysis of journal and co-cited journals reveals that Evidence-based Complementary and Alternative Medicine has the highest number of publications. The journal also ranks 4th in terms of citation count, indicating its significant contribution and dissemination of fMRI research in the field of acupuncture. Among the top 10 journals in terms of publication frequency, Neural Regeneration Research (IF = 5.9), Frontiers in Neuroscience (IF = 3.2), and Neural Plasticity (IF = 3.0) exhibit the highest impact factors. The majority of impact factors are below 5, and the JCR classification is mainly Q3, implying that fMRI research in acupuncture needs to be published in high-quality articles to enhance its influence. The journal with the highest number of citations is Neuroimage, which primarily falls into the Q1 and Q2 categories, indicating a superior level of quality and providing a solid foundation for fMRI research in acupuncture.

The analysis of journal and co-cited journals reveals that the most frequently published journal is Evidence-based Complementary and Alternative Medicine. Furthermore, this journal ranks fourth in terms of citation count, indicating that it has provided substantial support for research on fMRI in the field of acupuncture and has a certain level of dissemination impact. Among the top 10 journals by publication volume, the journals with the highest impact factors are Neural Regeneration Research (IF = 5.9), Frontiers in Neuroscience (IF = 3.2), and Neural Plasticity (IF = 3.0). Most of the journals have impact factors below 5, and the majority are categorized in the Q3 quartile according to JCR, suggesting that research on fMRI in acupuncture needs to be published in higher-quality journals to enhance its influence. The most frequently cited journal is Neuroimage, and the journals that are most frequently cited are primarily in the Q1 and Q2 quartiles, demonstrating their high quality and providing a solid foundation for research on fMRI in the field of acupuncture.

Co-citation analysis of references reveals that these cited studies are all clinical trials. The sample sizes of the included studies range from 10 to 36 participants, which, despite being relatively small, may be attributed to the high cost associated with fMRI. The most frequently cited reference is the 2008 article by Dhond et al. (2008), which demonstrated that acupuncture can enhance the spatial extent of resting-state brain networks, including brain regions related to pain modulation, memory processing, and emotional regulation. The second most cited reference is the article by Fang et al. (2009), which, through fMRI detection of real and sham acupuncture points, demonstrated that acupuncture leads to widespread deactivation of the limbic-paralimbic-neocortical system. The third and fourth most cited references are two articles by Bai LJ published in 2009. The third article Bai et al. (2009) demonstrated through fMRI findings that acupuncture can enhance the bipartite division of the anti-correlated resting-state networks and regulate the spontaneous activity of the intrinsic sensory-autonomic networks. The fourth article Bai et al. (2009) confirmed that brain activity patterns during rest are related to the type of stimulation. Significant stimulation-related activity was still present during the “rest” phase, particularly in the cerebellum and limbic brain regions. The fifth article Hui et al. (2005) used fMRI to explore how acupuncture at the Zusanli (ST36) point can modulate the neural activity of cortical and subcortical structures, including the cerebellum, diencephalon, and brainstem, at multiple levels. The highest centrality is held by the article by You et al. (2013), which used fMRI and MEG analysis to explore the specific biological mechanisms underlying acupuncture across spatial and temporal domains. This study found that modulation of the posterior cingulate cortex (PCC) within the theta (4–8 Hz), alpha (8–13 Hz), and gamma (30–48 Hz) frequency bands regulates the Default Mode Network (DMN) hub. The second most central reference is the article by Yeo et al. (2012), which used fMRI to show that acupuncture at GB34 (Yanglingquan) can increase neural responses in regions such as the substantia nigra, caudate nucleus, thalamus, and putamen, which are affected by Parkinson’s disease, confirming that acupuncture at GB34 may effectively improve symptoms of Parkinson’s disease. The third article is by Chen et al. (2015), which used fMRI to discover that acupuncture at GB34 can increase motor-cognitive connectivity while simultaneously reducing contralateral motor cortex compensation, thus promoting the rehabilitation of hemiplegia and spasticity. The articles by Chen et al. (2015), Li et al. (2010), and Wang et al. (2016) are tied for fourth. Chen et al. (2015) used fMRI to show that acupuncture can treat pain caused by knee osteoarthritis by enhancing functional connectivity between the right frontoparietal network (fFPN), the executive control network (ECN), and the descending pain modulation pathways. Li et al. (2010), conducted a multi-voxel pattern analysis (MVPA) based on fMRI data to explore the specificity of acupuncture at Guangming (GB37) and a nearby non-acupoint (NAP) on visual-related brain activation patterns. The study found that multiple brain regions could distinguish the central nervous response patterns caused by acupuncture at these two points, with the most significant areas being the occipital cortex, the limbic-cerebellar system, and subregions of the somatosensory cortex. Wang et al. (2016) conducted experiments that showed acupuncture can enhance resting-state functional connectivity between the left amygdala and the anterior cingulate cortex, as well as between the right amygdala and the left parahippocampal gyrus, thus helping to counter depression.

### 4.2 Research hotspots

By conducting keyword co-occurrence analysis and keyword clustering, the main research hotspots can be summarized as follows: different acupuncture techniques, acupoint specificity, and mechanisms of acupuncture in the treatment of diseases.

#### 4.2.1 Different acupuncture techniques

Common intervention methods include electroacupuncture, acupuncture, and high-tech acupuncture, which encompasses techniques such as balance acupuncture, auricular acupuncture, and wrist-ankle acupuncture. Electroacupuncture is the most frequently used method. FMRI studies have shown that electroacupuncture induces a broader range of activation changes (Kong et al., 2007). Vitaly Napadow et al. (2005) suggested that all acupuncture stimuli result in a signal reduction in the amygdala, anterior hippocampus, the cortices of the subgenual and retrosplenial, and ventromedial prefrontal cortex, while signals increase in the anterior insula. However, compared to manual acupuncture, electroacupuncture at low frequencies induces a larger area of fMRI signal increase, with significant signals only observed in the anterior mid-cingulate cortex. Acupuncture is a widely used intervention. Wang et al. (2020) utilized fMRI to study the efficacy of multi-point acupuncture for treating primary insomnia and its effects on brain activity. High-tech acupuncture mainly refers to laser acupuncture, which is considered a safe and reliable option (Beissner et al., 2011). Existing studies have used fMRI to observe the impact of laser acupuncture on brain activity when stimulating acupuncture points (Lv et al., 2016; Chang et al., 2023).

#### 4.2.2 Acupoint specificity

Stimulation of acupoints activates the brain Acupoints reflect the specificity of acupoints. Jung et al. (2015) found that both real acupuncture and fake acupuncture could activate the insula, anterior cingulate cortex, secondary somatic sensory cortex parietal cortex, and inactivate the medial prefrontal cortex, posterior cingulate cortex, inferior parietal cortex, and parahippocampal brain (Cao et al., 2019). Compared with shame acupuncture, real acupuncture showed greater brain activation in the posterior insula, posterior gill cover, and the tail of the anterior cingulate cortex. The study conducted by Ma et al. (2020) demonstrated the inhibitory effect of acupuncture on the marginal zone in rats with dysmenorrhea. Furthermore, acupuncture can modulate thalamocortical networks by enhancing connectivity between the mediodorsal thalamus (MD) and angular gyrus/posterior cingulate cortex/parahippocampal gyrus (PHG), as well as between motor thalamus subregions (Mthal) and medial prefrontal cortex (mPFC)/PCC/lateral temporal lobe cortical regions, while simultaneously reducing connectivity between MD and precuneus (PCU)/ middle cingulate cortex (MCC) (Kong et al., 2023). The findings of a review article suggest that acupuncture exerts an influence not only on the primary motor cortex and premotor cortex, but also leads to structural changes in language-related brain regions, including the inferior frontal gyrus, temporal lobe, occipital lobe, and parietal lobe (Zhang et al., 2021).

#### 4.2.3 Mechanisms of acupuncture in the treatment of diseases

Functional connectivity can be used to study the mechanisms of acupuncture-induced analgesia. Pain is a widespread and complex symptom, and many patients heavily rely on analgesics. However, analgesics often present issues such as safety concerns and the potential for addiction (Qiao et al., 2020). In contrast, acupuncture is well-tolerated and generates fewer adverse effects. Acupuncture can alleviate pain by stimulating specific acupuncture points on the body (Chen et al., 2020; Robinson et al., 2022), which has made it increasingly popular. Neuroimaging techniques are essential for exploring the central mechanisms of acupuncture analgesia, with fMRI being able to reveal multifunctional brain networks associated with pain perception (Damascelli et al., 2022). Additionally, functional connectivity analysis has been used to explore the relationships between brain regions, helping to better understand analgesic mechanisms (Lee et al., 2019). In a study by Xiang et al. (2021), it was noted that ankle acupuncture alleviated chronic low back pain symptoms by modulating the cerebellum and right insula. Yang et al. (2022) used fMRI to explore the mechanisms of moxibustion in alleviating pain in patients with primary dysmenorrhea and found that moxibustion significantly increased the resting-state functional connectivity (rs-FC) of the left inferior frontal gyrus (IFG), bilateral anterior cingulate cortex (ACC)/middle cingulate cortex (MCC), left posterior cingulate cortex (PCC)/precuneus (PCU), and left parahippocampal gyrus (PHG). Wang et al. (2021) used fMRI to investigate the immediate analgesic effects of acupuncture on patients with primary dysmenorrhea, discovering that acupuncture significantly reduced the resting-state functional connectivity between the rostral anterior cingulate cortex (rACC) and left precentral gyrus. Wei et al. (2022) used fMRI to analyze changes in functional connectivity in patients with migraine without aura. The results showed that electroacupuncture at the rate of shuaigu (GB80) could modulate the functional connectivity between the right insular subregion and the parietal lobe, specifically the right dorsal anterior insula and the right postcentral gyrus, as well as between the right posterior insula and the left precuneus, thereby improving symptoms in patients with migraine without aura.

Neural regeneration is an important central mechanism for studying brain-related diseases. The regeneration of the nervous system necessitates the repair or replacement of impaired nerve cells resulting from injury or disease (Steward et al., 2013). Ischemic stroke is a sudden neurological deficit resulting from the cessation of blood flow to a specific region of the brain (Haupt et al., 2023). While acupuncture can stimulate neurogenesis, activate axonal regeneration and sprouting, improve synaptic structure and function, and enhance neural plasticity (Qin et al., 2022), especially when using appropriate electroacupuncture parameters for treatment, it can play a role in neuroprotection and promoting nerve regeneration (Chang et al., 2018). The loss of neurons and synapses is a primary pathological feature of cognitive impairment. Stimulating acupoints can restore brain functional activity and connectivity, promote neuroregeneration, and regulate synaptic plasticity to facilitate the formation of normal neural circuits (Zhang et al., 2023). FMRI can assess spontaneous neuronal activity through measures such as ALFF (Amplitude of Low-Frequency Fluctuations) and ReHo (Regional Homogeneity) (Zang et al., 2023). Changes in ReHo values can serve as one of the neurobiological markers reflecting disease (Zhang et al., 2018). Research by Li et al. (2022) demonstrated that electroacupuncture at Zusanli (ST36) and Quchi (LI11) significantly increased ReHo values in the ipsilateral posterior hippocampus, contralateral anterior hippocampus, contralateral subhippocampus, contralateral dorsolateral thalamus, ipsilateral somatosensory cortex, and ipsilateral substantia gelatinosa area, thereby promoting the activation of neurons in brain regions associated with cognition and behavior, reducing neuronal death. According to a comprehensive summary by Yin et al. (2023), acupuncture for cognitive disorders, through stimulation of 10 key brain areas such as the cingulate cortex, middle frontal gyrus, and hippocampus, can effectively promote neuroplasticity and facilitate repair. Zhang et al. (2024) utilized fMRI to investigate the underlying mechanism of acupuncture treatment for mild cognitive impairment (MCI). The findings revealed that following acupuncture intervention, MCI patients exhibited contrasting alterations in ReHo values within the hippocampus/parahippocampal and insular regions compared to baseline, while a notable increase in ReHo values was observed within the middle frontal gyrus region. These results suggest that acupuncture has the potential to modulate brain activity, particularly within the default mode network and salience network, thereby facilitating cognitive enhancement in individuals with MCI.

### 4.3 Research trend analysis

The emergence of keywords to a certain extent reflects the prevailing research trends. The graph reveals that keywords such as randomized controlled trial, meta-analysis, functional connectivity, ischemic stroke, women, systematic review, prophylaxis, mild cognitive impairment, and others have consistently maintained their popularity in current research directions.

#### 4.3.1 Randomized controlled trials and functional connectivity

From the perspective of evidence-based medicine, well-designed randomized controlled trials (RCT) provide the essential information and guidance required for clinical practice decisions and are considered the gold standard, sitting at the top of the evidence pyramid. RCT is the most rigorous and reliable research method to determine whether there is a causal relationship between interventions and outcomes (Li et al., 2018; Bhide et al., 2018). Although fMRI has made significant progress in studying the safety and efficacy of acupuncture for treating diseases, the central mechanisms remain unclear and require further investigation. RCT is the best approach for this purpose. Functional connectivity can be used to explore the connectivity and interactions between brain regions within brain networks during specific stimuli (Xie et al., 2014; Liu et al., 2015). Therefore, using RCTs to explore brain functional connectivity can help reveal the underlying central mechanisms. Several RCT has shown (Liu et al., 2022; Li et al., 2022) that acupuncture treatment can enhance functional connectivity between specific regions when treating conditions such as anxiety and migraines, and that different doses of laser acupuncture lead to changes in functional connectivity between the regions of interest (ROI) and other brain areas (Chang et al., 2023). Additionally, an ongoing RCT is exploring the effectiveness and safety of acupuncture in treating post-stroke depression, particularly focusing on its impact on the functional connectivity of the cognitive control network (CCN) (Luo et al., 2022).

#### 4.3.2 Meta-analysis and systematic review

Meta-analysis is a method that synthesizes, combines, and analyzes data from multiple studies to estimate a single effect and answer research questions (Arya et al., 2020), while systematic reviews are considered the gold standard for evidence synthesis (Paul and Leibovici, 2014). The literature included in this article related to meta-analysis and systematic reviews predominantly focuses on using fMRI to systematically summarize the mechanisms of acupuncture in treating brain disorders. Research by Zang et al. (2023) showed that fMRI revealed significant effects of acupuncture on the default mode network in patients with primary insomnia, particularly in the frontal and precuneus regions. A study by Zhang et al. (2021) demonstrated that after acupuncture treatment for depression, fMRI showed an increase in the N-acetylaspartate/creatine (NAA/Cr) ratio, increased ALFF in the right precuneus, decreased ALFF in the inferior frontal gyrus (IFG), and enhanced functional connectivity in the anterior cingulate cortex (ACC).

#### 4.3.3 Ischemic stroke and women

Ischemic stroke is one of the most common and severe manifestations of cerebrovascular disease (Feske, 2021). Clinically, it presents as focal neurological deficits such as hemiplegia, aphasia, dysphagia, visual impairment, and cognitive impairments (Li et al., 2022). The incidence of stroke ranks second among the leading causes of death worldwide, accounting for 11.6% of all mortalities, and it also stands as the third most prevalent cause of disability globally, affecting 5.7% of the population (GBD 2019 Stroke Collaborators, 2021). Compared with males, females with ischemic stroke exhibit accelerated physiological aging, especially among younger populations where the incidence of ischemic stroke is higher in females than in males (Gallego-Fabrega et al., 2022; Ekker and de Leeuw, 2020). Additionally, females have a higher lifetime risk of developing ischemic stroke compared to males (Bruce et al., 2020). Acupuncture can promote neuroplasticity by modulating the functional reorganization of the entire brain after ischemia and by altering neural structure and function (Qin et al., 2022; Chavez et al., 2017) and has been used in the treatment of stroke and post-stroke rehabilitation (Lu et al., 2016). The main analysis methods utilized in fMRI are ReHo and ALFF, which have been extensively employed in investigating brain function and neural plasticity following ischemic stroke (Li et al., 2022; Lv et al., 2021). The findings of an fMRI-based study (Wang et al., 2023) demonstrate that acupuncture has the potential to enhance motor function following ischemic stroke. The results of animal experiments have demonstrated that electrical acupuncture stimulation applied to the GV20 and DU24 acupoints can significantly enhance the levels of N-acetyl aspartate (NAA) and choline (Cho) in both the hippocampus and prefrontal cortex, thereby effectively improving learning and memory abilities in rats with ischemic stroke (He et al., 2018).

#### 4.3.4 Mild cognitive impairment and prophylaxis

MCI is a disease in which individuals exhibit cognitive impairments with minimal impact on daily activities. The prevalence of MCI in the age group of 60–64 years is 6.7%, whereas it increases to 8.4% in the age group of 65–69 years, exhibiting an ascending trend with advancing age (Petersen et al., 2018). MCI can be regarded as the primary cognitive manifestation of Alzheimer’s disease (AD), but it may also occur in other diseases such as Parkinson’s disease, Huntington’s disease, and human immunodeficiency virus (Sanford, 2017). Therefore, effectively treating MCI can prevent the onset and progression of AD and other related diseases. The practice of acupuncture is widely recognized as an effective medical system that not only provides treatment for various diseases but also offers preventive measures for maintaining optimal health (Mallory et al., 2016). Task-based fMRI can enable the assessment of pre-and post-acupuncture treatment changes in brain activity (Yan et al., 2022), while resting-state fMRI measures spontaneous neuronal activity through the recording of blood oxygen level-dependent BOLD signals (Zhao et al., 2023). Thus, fMRI can be applied to investigate the development of acupuncture for the prevention of MCI. Xu et al. (2022) using fMRI during acupuncture treatment for MCI patients, observed a significant voxel-wise centrality increase in bilateral medial frontal cortex (MFC) and bilateral MCC after acupuncture stimulation, along with decreased voxel-wise centrality in left precuneus region. This effectively enhanced patients’ cognitive function and mitigated the progression of MCI. When Lai et al. (2022) explored the efficacy and mechanism of moxibustion treatment for patients with MCI using fMRI, they observed a significant increase ALFF values in the left precuneus, left thalamus, and right temporal pole after moxibustion therapy. Additionally, there was a significant decrease observed in the ALFF values within the bilateral lingual gyrus. This improvement in cognitive function prevented further deterioration of MCI patients.

### 4.4 Analysis of FMRI in the field of acupuncture

From the above analysis, it can be observed that while the number of annual publications on fMRI in the field of acupuncture fluctuates, there is an overall upward trend. The countries and institutions with the highest publication volumes are primarily from China, the USA, and South Korea, with fewer studies emerging from other countries. Unfortunately, based on the journals in which the articles are published, research on fMRI in acupuncture appears primarily in a few high-quality journals, with most journals having a lower impact factor. Our study reveals that the current research hotspots mainly focus on different acupuncture techniques, the specificity of acupoints, and the mechanisms of acupuncture in treating diseases. Furthermore, we have predicted future research trends. The results indicate that studying fMRI in acupuncture from the perspective of evidence-based medicine is a growing trend, as it can provide high-quality evidence. Additionally, there is a clear need for more meta-analyses and systematic reviews to summarize the application of fMRI in the acupuncture field. Moreover, our findings also suggest that fMRI research in acupuncture includes studies on the reasons why women have a higher incidence of ischemic stroke compared to men, as well as its role in preventing mild cognitive impairment.

**Figure 8:**
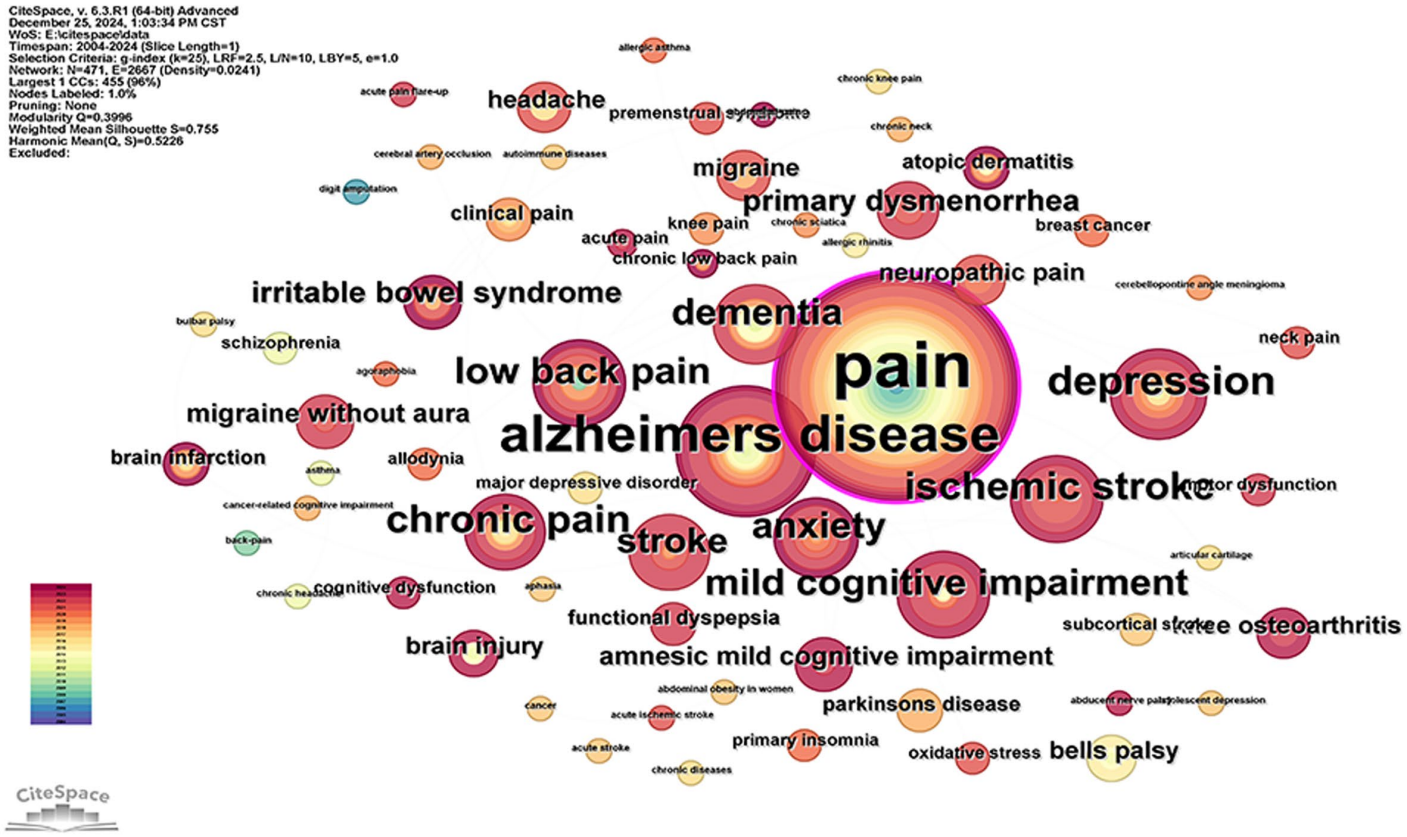
List of diseases.

FMRI has been used in the field of acupuncture for a wide range of disorders, focusing on motor system disorders, neurological disorders, and mental disorders (Figure 8). Disorders of the locomotor system include chronic pain, low back pain, neck pain, knee pain, carpal tunnel syndrome, motor dysfunction, and more! Neurological disorders include headache, migraine, abducent nerve palsy, bulbar palsy, bells palsy, dementia, aphasia, mild cognitive impairment, cancer-related cognitive dysfunction, cerebral arterial occlusion, and stroke. Mental disorders include primary insomnia, anxiety, depression, and schizophrenia. All of these illustrate the variety and wide range of applications of using fMRI to study acupuncture for the treatment of diseases.

## 5 Advantages and limitations

Zhang et al. (2021) have summarized the history and research hotspots of acupuncture in the field of MRI. The similarities between his study and the present study are that the country with the highest number of annual publications is China and the author with the highest number of publications is Tian, J. The journal with the most publications is Evidence-based Complementary and Alternative Medicine. The highest co-cited journal frequency was Neuroimage. The keywords are similar in that they all have fMRI, Pain Acupuncture, etc. The difference is that his findings show that the trends lie in Connectivity, fMRI, Modulation, and DMN. The results of this study show trends include the randomized controlled trial, meta-analysis, functional connectivity, ischemic stroke, women, systematic review, prophylaxis, and mild cognitive impairment.

This study employs bibliometric methods to visualize the application of fMRI in the field of acupuncture from 2004 to 2024, summarizing the current research status and predicting future trends. However, it is important to note that there are certain limitations in this study. First, we relied solely on the WOS database as the data source, which may have excluded some relevant literature. Second, all the studies included were published in English, without considering literature in other languages. Third, during the exclusion process, some types of literature that belonged to two categories were not fully excluded, which may have affected the thoroughness of the analysis.

## 6 Conclusion

This study aims to explore the application of fMRI in the field of acupuncture from 2004 to 2024. It provides an overview of current research status and offers predictions for future trends. The current research suggests that fMRI has a broad range of applications in the field of acupuncture and exhibits significant potential for further development. Despite the limitations of this work, the results may help researchers studying fMRI and acupuncture. We predicted future research trends. We believe that studying the application of FMRI in the field of acupuncture from the perspective of evidence-based medicine is the future trend and that high-quality evidence can be obtained from the perspective of evidence-based medicine. More Meta-analyses and systematic evaluations are also needed to conclude the application of fMRI in the field of acupuncture. In addition, we believe that the application of fMRI in the field of acupuncture also includes the reasons why women get ischaemic stroke higher than men, and the prevention of mild cognitive impairment.
